# Translating medicines to patients: A novel methodology for quantifying the global medical supplies and donations program

**DOI:** 10.1371/journal.pone.0206790

**Published:** 2018-11-02

**Authors:** Shang-Ju Li, Elisabeth Vodicka, Anne Peterson, Andy Stergachis

**Affiliations:** 1 AmeriCares Foundation, Inc., Stamford, Connecticut, United States of America; 2 University of Washington, School of Pharmacy, Seattle, Washington, United States of America; 3 University of Washington, School of Public Health, Seattle, Washington, United States of America; Public Library of Science, UNITED KINGDOM

## Abstract

**Background:**

International medical donation programs can help alleviate the burden of illness and serve as a safety net for the global health care system. However, to our knowledge no studies have assessed the number of individuals served through medical donation programs. As such, this study aimed to evaluate the impact of the Americares Foundation’s (Americares) medical donation program in terms of the number of patients served.

**Methods:**

We conducted an outcome evaluation study in 34 health facilities in 10 countries that receive medical donations from Americares. Medical records were randomly sampled at each participating facility and evaluated for types of medications and number of courses of prescribed treatments. Facility level data and donation inventory data were also collected. We developed an algorithm for converting quantities of donated medicines into the number of individuals served at the facility level. These estimates were then extrapolated to the country and region levels to assess the total impact of medications donated in 2015. Probabilistic sensitivity analysis was conducted to derive 95% credible ranges for projected estimates and to assess model uncertainty.

**Results:**

Records of 3,205 unique patients were reviewed, encompassing 10,449 medical visits. The average number of medications and courses of treatments prescribed per visit were 2.63 and 2.68, respectively. The average medication destruction rate ranged from 0% to 24% at facilities, with a cross-country average of 7%. For the 10 countries included in the study, we project that 700,377 unique individuals were served through the program (95% credible range: 518,401–905,982). Scaled across all regions receiving Americares donations, we project that the program supported an estimated 5.1 million beneficiaries, including 484,188 chronic care and 4.65 million acute care patients.

**Conclusions:**

This study provides a novel methodology for medical donation programs seeking to estimate one of their key outcomes—patients served—and global reach. Rigorous assessments of program outcomes can provide important insights into the value of medical donation initiatives.

**Trial registration:**

Human subjects approval was received from the University of Washington Institutional Review Board (Approval #52316; 7/19/2016).

## Introduction

Medicines and medical supplies are essential commodities for improving health. International medical donations can help to alleviate the burden of illness and serve as a safety net for the global health care system and decrease health care costs for low-resourced health systems. Donations from for-profit and non-governmental organizations to Ministries of Health and non-governmental health organizations in low-and-middle income countries (LMICs) can fill medication gaps for communities where access to medicines is limited due to costs, other barriers to access, humanitarian crises, natural disasters and other emergency settings.[1] However, it is necessary to ensure that donated medications are high quality, safe, and relevant to local needs. As such, the World Health Organization established guidelines for medical donations and surveillance to support coordinated efforts between donors and recipient countries that optimize the “effectiveness, efficiency and adequacy” of donations.[2] The guidelines emphasize the need for monitoring and evaluation of donation programs; yet, few evaluations have been reported assessing the outcomes of medical donation programs. [3–7]Such evaluations provide important insights about the value of these initiatives.

The Americares Foundation (Americares) is a humanitarian and global health organization that endeavors to save lives and build healthier futures for people in crisis in the U.S. and around the world. Each year, Americares delivers more than $700 million in donated medicines and supplies through its *Access to Medicines* program to more than 40 local distribution partners to reach an estimate of 3,200 health facilities, Ministries of Health and social service organizations serving low-income communities in the U.S. and globally. Requests for donations are submitted to Americares by potential recipients, after which Americares and the distribution partners coordinate the shipment to local recipients. Product information such as generic name, brand name, expiration date, and quantities–in terms of numbers of pills and course treatments–are documented by Americares, while the distribution partners maintain a database of available priority medicines, supplies, and equipment, as well as capacity constraints of potential donation recipients. Systems for monitoring and evaluating donations–including donated products and supply chain systems, prescribing patterns, and individual use of medicines–becomes critical to ensure that course treatments are appropriately allocated to facilities and patients in need.[8]

Prior research on medical donations has quantitatively and qualitatively assessed programs aimed to improve monitoring of medical donation expiry and adequacy of facility-level dispensation.[9] However, to our knowledge no studies have assessed the number of individuals served through medical donation programs. As such, this study aimed to quantify the number of patients supported by Americares' donated medications received through health facilities distribution or self-procurement by local healthcare facilities.[10] In doing so, the study also aimed to develop a standardized algorithm for converting donated medications into course treatments. This metric can provide other donors and local in-country healthcare partners with methods to assess the impact of their programs at the patient-level. It is our hope that its use will inform decisions related to how medications and supplies are donated to maximize the population health outcomes of these donations.

## Methodology

We developed a two-step methodology for estimating the number of medicines donated to the number of individuals served by converting quantities of tablets and vials donated into course treatments and converting course treatments into individuals. We define one course treatment as the standard amount of medicine used to treat a patient with a specific medical condition for a specified time period. This is based on the most common treatment regimen for a specific dose, formulation and packaging prescribed to patients. For example, clarithromycin is commonly used as a treatment for many types of lower respiratory tract infections. The average adult daily regimen is 500 mg twice daily, and the average duration of treatment is 10 days. Therefore, 20 tablets of clarithromycin 500 mg equals to one course treatment. All course treatments were determined by Americares Medical Units composed of licensed physicians and pharmacists (described in detail in S1 Appendix). Individuals, especially those on medications for chronic disease and those with multiple diagnoses, commonly receive more than one course treatment.

**Fig 1:**
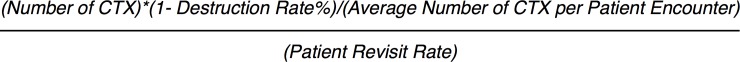
Americares course treatments to beneficiaries algorithm*. * CTX = Courses of treatment. One course treatment is defined as the average amount of medicine used to treat a patient with a specific medical condition for a specified time period, based on the most common treatment regimen for a specific dose, formulation and packaging prescribed to patients.

As a result, the number of course treatments donated does not translate directly to the number of patient treated. Therefore, we developed an algorithm to convert donated course treatments into an estimated number of patients treated (Fig 1; additional calculations used are shown in S2 Appendix). The numerator is the number of course treatments prescribed and used, excluding any wasted or destroyed drug, divided by the average number of course treatments per patient visit (as defined by the drug prescribing indicator developed by the WHO and International Network for the Rational Use of Drugs).[11] It provides an estimate of the number of patient visits resulting in an actual prescription supported by the donated course treatments. This number is then divided by the average frequency with which patients revisit the facility to obtain an estimate of the number of patients served. The reciprocal of the average number of course treatments per visit multiplied by the revisit rate is the “conversion factor” in our study for translating medications to beneficiaries.

It is important to note the difference between “unique patients” and “total beneficiaries” to avoid underestimating the potential impact of medicine donations. We define unique patients as the unduplicated number of patients receiving course treatments. However, it is likely for patients with co-morbidities to receive multiple medications for different diseases. We define the total beneficiaries as the total number of diseases treated with donated medications. Therefore, a unique patient could receive health benefits from more than one medicine.

We first conducted a pilot study with a sample of 100 patients attending an Americares-managed clinic in Connecticut to validate the algorithm. The clinic selected for the pilot has an electronic medical record system that allowed for independent tracking of unique patients entering the health system, as well as algorithm variables such as patient-level revisit rates, prescriptions, and course treatments per patient visit by disease type. We then conducted a secondary analysis to estimate the total number of beneficiaries across all Americares donation sites. Data for the analysis was derived from two sources: (1) Americares’ inventory tracking system and (2) prescription and medication refill records from a sample of local healthcare partners collected as part of an Americares’ evaluation of their program conducted in June-October 2016. The inventory tracking system contains every medication’s destination country, facility name and location, medication name, average course treatments, shipment date and treatment type (acute/chronic). Inventory data was extracted on all medications shipped to a healthcare partner between January 1 and December 31, 2015. Ten countries were then selected for participation in the Americares evaluation, based on number of course treatment donations received, as well as discussions between Americares and the local country director and partners regarding feasibility, accessibility and safety of undertaking primary data collection for the evaluation exercise. The countries included were: El Salvador, Ghana, India, Nicaragua, Peru, Philippines, Romania, Tanzania, United States, and West Bank. The five health facilities in each country that received the largest volumes of donated medicines in 2015 were then included in the evaluation.

De-identified data were obtained for a sample of 100 patients at each participating clinic who were prescribed medications during the evaluation period using systematic sampling methods.[12] The data collector randomly chose a starting number for sampling within the first interval of the total number of active patients on the clinical panel. Patient-level variables collected included date of visit, disease type (chronic or acute), and number of medications and course treatments prescribed. Facility-level variables included the medication destruction rate (% of medications destructed, expired, or unused), facility name, city, country and state, level of facility (i.e., primary healthcare clinics, secondary care center, tertiary care center, and medical outreach posts), total number of patients at the facility, and total number of visits at the facility. Inventory data was provided for information about donated medications at the country level (i.e., total number of medications donated, stratified by chronic and acute medications; level of facilities receiving donations).

## Analysis

Our first level of analysis used data from the pilot study to evaluate the ability of the algorithm to accurately estimate, within a 95% credible range, the number of unique patients that benefit from Americares’ medication donations within a given clinic. According to the inventory system, the Americares-managed clinics received 13,319 course treatments in 2015. In the pilot clinic, 288 course treatments were prescribed across 100 patients during a total of 843 facility visits. It was estimated that no drug wastage occurred. With these parameters, the algorithm yielded an estimate of 4,633 patients benefiting from medication donations. However, the clinic’s EMR data indicated that the actual number of unique patient beneficiaries was 3,071. A one-way sensitivity analysis was conducted to evaluate the influence of each algorithm variable on the mathematical output. We found that the estimated number of patient visits at the facility and assumptions about wastage (e.g., destruction rate) had the greatest influence on algorithm output. Since the number of patient visits was known via the EMR system, we revised our estimates of drug wastage to reflect the average estimated destruction rate across the U.S. Americares study sites (24%) and allowed for variation across a minimum and maximum range (0%-40%). With this revised assumption, our algorithm estimated that 3,512 unique patients benefited from medication donations at the clinic, with a 95% credible interval encompassing the actual estimate of 3,071 (95% credible interval: 2810, 4215).

We then conducted the secondary analysis in a two-step process: (1) analysis of the sample facility-level data; and (2) projection of country- and region-level estimates based on extrapolations of the facility-level data. First, we performed descriptive analyses of the facility-level data to obtain the unique number of patients, mean number of visits, and mean number of medications and course treatments prescribed among our sample population. Analyses for each country were conducted for the total population and stratified by disease type (chronic vs. acute). These estimates were used in the Americares’ algorithm to calculate the total number of beneficiaries served in the sample population. Descriptive analyses were conducted using Stata 13 (StataCorp. 2013. Stata Statistical Software: Release 13. College Station, TX: StataCorp LP.).

Second, we used the means and standard errors derived from the sample data analysis to project the potential number of patients, visits, medications and course treatments prescribed, and total number of Americares’ beneficiaries at the country and region levels. A probabilistic sensitivity analysis was conducted to derive 95% credible interval for the projected country- and region-level estimates and evaluate uncertainty in the model. We used a Monte Carlo simulation that varied each of the model parameters simultaneously. The Monte Carlo assumed the estimate derived from the sample data as the base case and then re-ran the model 10,000 times using a value for each parameter randomly drawn from its assumed distribution (beta distribution for probabilities and normal distribution for all other parameters). Standard errors were used when available from the facility-level sample data to calculate the plausible range for the distributions. Where these were unavailable (e.g., destruction rates), we varied the parameters +/-20% from the base case values. All projections were conducted using Microsoft Excel (Renton, WA; 2016).

Certain assumptions were made to extrapolate to the country and region levels. First, we applied the average destruction rates, patient revisit rates and average number of course treatments prescribed per visit across sample facilities to calculations for the country. At the region-level, we created a weighted average for these variables based on average facility-level estimates from each country in the region. Additionally, we applied a weighted distribution of the level of facilities receiving donations in each country to the region level to assess the proportion of beneficiaries served across facility levels. For example, the total number of facilities receiving donations in the Philippines was unknown, so we assumed that medical donations were distributed across the WHO reported number of public healthcare facilities (N = 701).[13] Population estimates for the country and region were derived from World Bank data.[14]

Americares completed the primary data collection as part of their ongoing internal monitoring and evaluation efforts. Ethics approval for secondary data analysis was received from the University of Washington Institutional Review Board. The chief medical officer and safety committee at each facility provided verbal informed consent prior to the Americares review of medical records.

## Results

**Fig 2:**
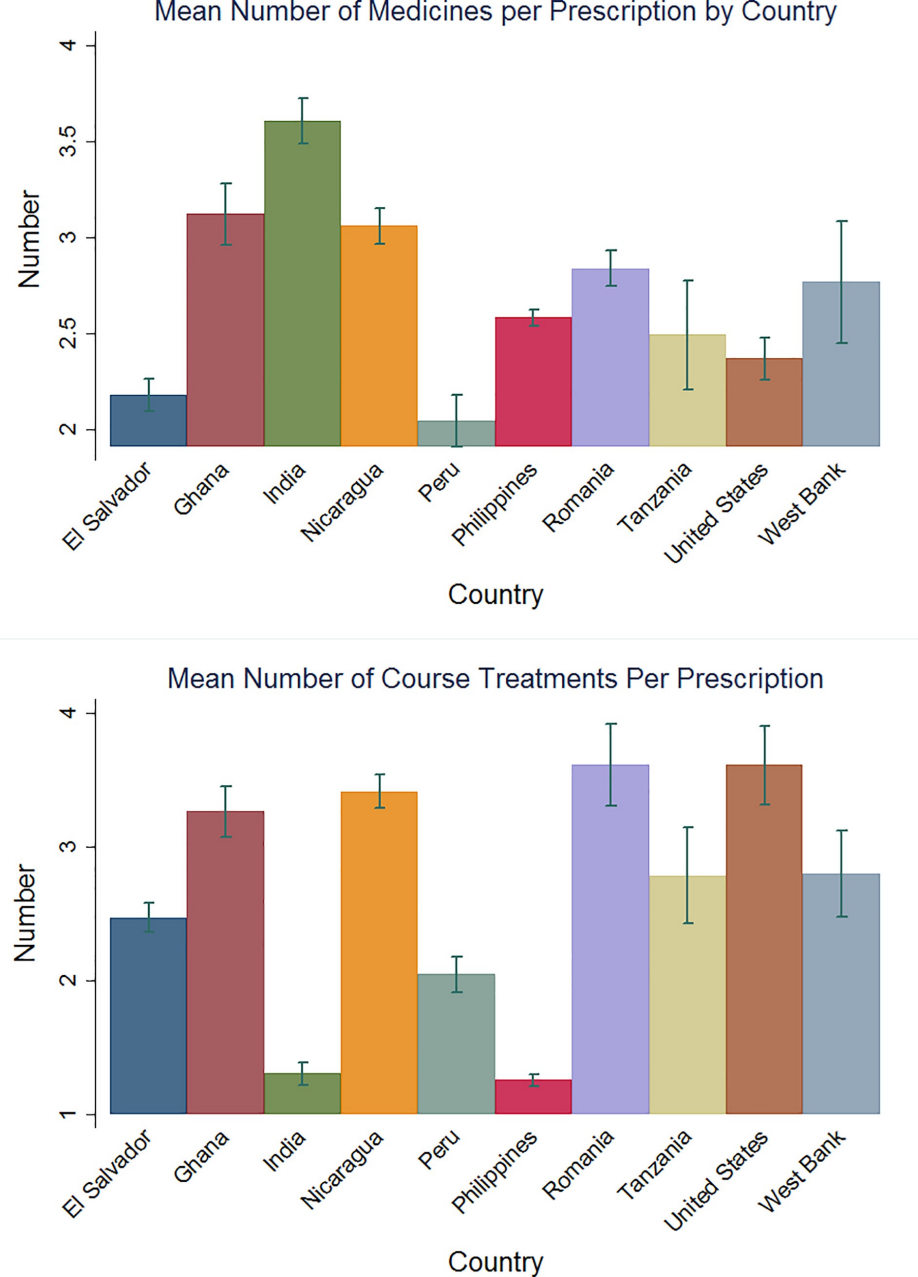
Estimated number medications and course treatments per prescription by country.

**Fig 3:**
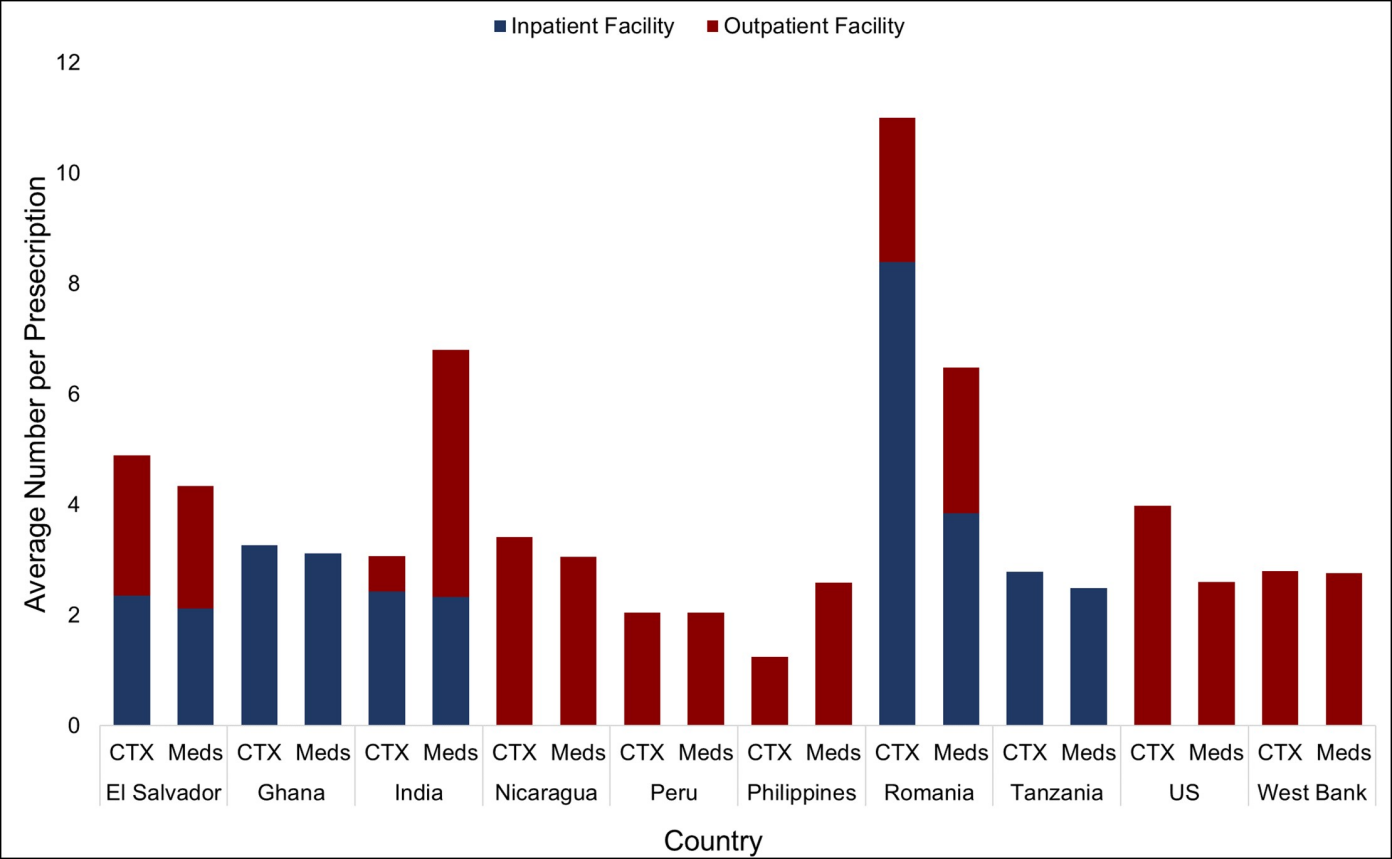
Estimated number medications and course treatments per prescription by level of care. *CTX = Average number of course treatments per prescription **Meds = Average number of medications per prescription.

**Fig 4:**
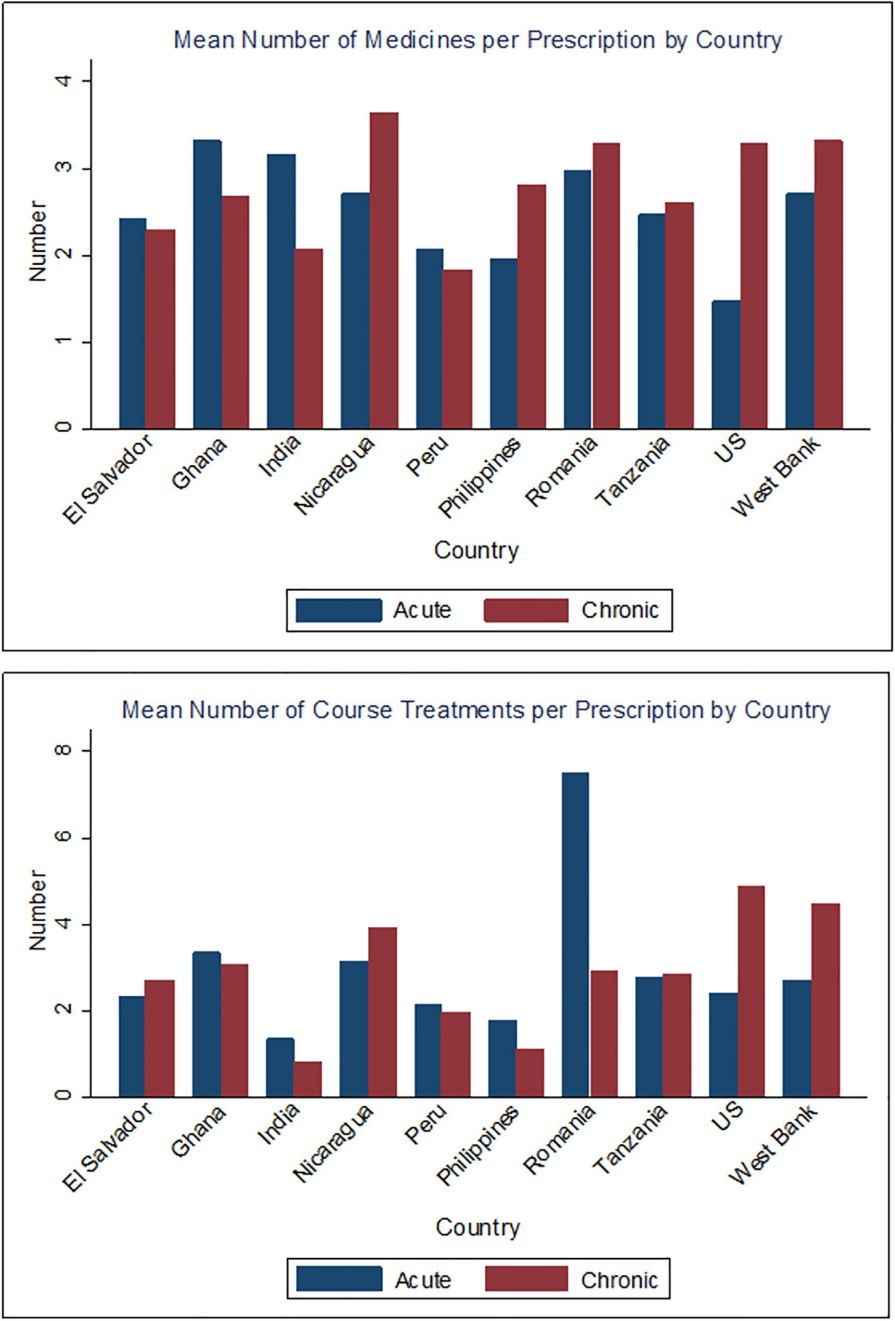
Estimated number medications and course treatments per prescription by chronic and acute conditions.

De-identified medical record data of 3,205 unique patients was included in the analysis, representing 10,449 total visits to the 34 health facilities evaluated. Two-thirds of the patients attended the clinics for acute conditions (67%, N = 2,471). On average across the 10 countries, 2.68 medications were prescribed per patient (95% confidence interval: 2.11, 3.16) and 2.63 course treatments were prescribed per patient (95% confidence interval: 2.14, 3.21). (Fig 2) The majority of medications and course treatments were provided in outpatient facilities. (Fig 3) We estimated that within the participating study facilities, nearly 73,000 unique patients (95% CI: 58,205–87,308) and 105,000 total beneficiaries (95% CI: 84,242–126,635) were served from the approximately 353,308 medications provided through Americares in 2015. Of the medications and course treatments prescribed during visits, 25% were chronic care and 75% were acute care patients. (Fig 4) The average medication destruction rate ranged from zero to 24% at sample facilities, with a cross-country average of 7%. (Table 1)

**Table 1:** Characteristics of study clinics by country.

Country	Total # of Visits	Total # of Unique Patients	# Unique Chronic Care Patients*	# Unique Acute Care Patients	# of Medications Prescribed per Patient Mean (95% CI)	# of CTXs** Prescribed per Visit Mean (95% CI)	Average Medicine Destruction (% Destroyed)
El Salvador	1395	378	139	297	2.18 (2.09, 2.27)	2.47 (2.38, 2.56)	16%
Ghanaⱡ	409	300	89	225	3.12 (2.96, 3.28)	3.26 (3.08, 3.45)	2%
India	647	284	34	268	3.61 (3.49, 3.73)	1.30 (1.22, 1.39)	4%
Nicaragua	1236	405	126	378	3.06 (2.97, 3.15)	3.41 (3.29, 3.54)	5%
Peruⱡ	402	200	122	162	2.05 (1.92, 2.18)	2.05 (1.91, 2.18)	10%
Philippines	2213	373	171	241	2.58 (2.54, 2.62)	1.25 (1.21, 1.29)	6%
Romania	1566	315	141	193	2.84 (2.75, 2.93)	3.62 (3.31, 3.92)	3%
Tanzania	261	96	15	89	2.49 (2.21, 2.77)	2.79 (2.47, 3.18)	2%
U.S.	2102	638	348	419	2.37 (2.26, 2.48)	3.61 (3.39, 3.32)	24%
West Bankⱡ	218	216	15	199	2.77 (2.45, 3.09)	2.80 (2.48, 3.12)	2%
**Total**	**10,449**	**3,205**	**1,200**	**2,471**	**2.68 (2.11, 3.16)**	**2.63 (2.14, 3.21)**	**7%**

*Note: Unique chronic care patients and unique acute care patients may not equal the total number of unique patients, since patients could attend the clinic for both a chronic and acute condition. They would therefore be included in the unique counts of each disease type, but only counted once in the total number of unique patients.

**CTX = courses of treatments

ⱡGhana, Peru and West Bank reported medicine destruction rates of 0%. To ensure a conservative approach that does not overestimate the benefit of medication donations, our analysis assumes that at least some amount of medication would be wasted at each facility. Therefore, for these countries, we applied the regional average destruction rate from data provided in other countries. For Ghana, we applied the average destruction rate from Tanzania. For Peru, we applied the average destruction rate from Nicaragua and El Salvador. West Bank is the only country from the Middle East & North Africa region, so we applied the average destruction rate from Tanzania

Extrapolated to the country level, we estimate that 2,225 facilities received 6.74 million course treatment donations across the 10 countries evaluated. Facilities in the United States received the most donations (39%), followed by El Salvador (28%) and the Philippines (13%), distributed over an estimated 2,203 health facilities (N = 984, N = 338, and N = 701, respectively). Ghana, India, and Romania received the lowest proportion of course treatments (0.8%, 0.6%, and 0.6% respectively), distributed over an estimated 46 clinics (N = 19, N = 16, and N = 11, respectively). Across the countries, 31% of course treatments donated were for chronic conditions vs. 69% for acute conditions. Our country-level projections estimate that approximately 700,000 unique patients were served across the 10 countries evaluated (95% credible interval: 518,401–905,982). As mentioned previously, patients could attend the clinic for more than one condition. Treatment of each condition would translate to treatment of one beneficiary. Extrapolating from the facility-level sample data to the ten countries evaluated, we project that the donation program yielded 1.95 million total beneficiaries (95% credible interval: 1,533,786–2,392,717), 181,665 chronic beneficiaries (95% credible interval: 133,436–237,762), and 1.76 million acute beneficiaries (95% credible interval: 1,378,517–2,201,927). (Table 2)

**Table 2:** Course treatment donations and projected number of beneficiaries served by country.

Country	# of Facilities Receiving Donations N (%)	Total CTX Donated N (%)	Chronic CTX Donated N (% of Total)	Acute CTX Donated to Country N (% of Total)		Total Beneficiaries N (95% CI)		
					Unique Patients Served N (95% CI)		Total Chronic Care Beneficiaries N (95% CI)	Total Acute Beneficiaries N (95% CI)
El Salvador	338 (15.2%)	1,857,380 (27.6%)	1,774,100 (95.5%)	83,280 (4.5%)	173,347 (128,257, 215,826)	665,562 (530,377, 813,479)	5,525 (3,994, 7,062)	660,036 (524,850, 807,623)
Ghana	19 (0.9%)	56,049 (0.8%)	42,190 (75.3%)	13,859 (24.7%)	12,341 (9,256, 16,219)	20,632 (16,982, 24,880)	3,382 (2,467, 4,513)	17,250 (13,566, 21,523)
India	16 (0.7%)	37,739 (0.6%)	25,844 (68.5%)	11,896 (31.5%)	11,549 (8,474, 15,450)	30,022 (24,494, 36,561)	11,690 (8,064, 17,031)	18,332 (14,507, 22,510)
Nicaragua	102 (4.6%)	657,553 (9.8%)	534,132 (81.2%)	123,422 (18.8%)	59,715 (45,716, 75,790)	169,533 (135,564, 201,124)	8,017 (6,076, 10,106)	161,516 (127,944, 194,107)
Peru	27 (1.2%)	208,164 (3.1%)	110,867 (53.3%)	97,297 (46.7%)	45,378 (32,918, 60,538)	81,688 (68,007, 99,033)	34,493 (23,927, 47,454)	47,195 (37,027, 57,441)
Philippines	701 (31.5%)	902,378 (13.4%)	702,467 (77.8%)	199,912 (22.2%)	105,992 (78,167, 138,259)	402,486 (324,763, 479,158)	23,880 (17,884, 31,018)	378,605 (299,627, 456,098)
Romania	11 (0.5%)	37,371 (0.6%)	18,697 (50.0%)	18,674 (50.0%)	2,136 (1,549, 2,844)	3,904 (3,168, 4,961)	1,469 (1,043, 2,052)	2,436 (1,801, 3,299)
Tanzania	4 (0.2%)	93,573 (1.4%)	73,788 (78.9%)	19,786 (21.1%)	12,109 (8,333, 17,503)	27,995 (21,848, 35,185)	1,895 (1,227, 2,848)	26,100 (20,090, 33,372)
U.S.	984 (44.2%)	2,598,043 (38.6%)	1,138,266 (43.8%)	1,459,777 (56.2%)	177,237 (134,199, 223,571)	442,696 (347,189, 571,629)	82,662 (62,531, 103,943)	360,034 (266,306, 486,345)
West Bank	23 (1.0%)	289,849 (4.3%)	250,416 (86.4%)	39,433 (13.6%)	100,574 (71,537, 139,987)	102,140 (81,397, 126,709)	8,652 (6,228, 11,738)	93,489 (72,803, 119,613)
**Total**	**2,225** **(100%)**	**6,738,099** **(100%)**	**4,037,836** **(60%)**	**2,757,600** **(40%)**	**700,377** **(518,401, 905,982)**	**1,946,659** **(1,553,786, 2,392,717)**	**181,665** **(133,436, 237,762)**	**1,764,994** **(1,378,517, 2,201,927)**

At the region-level, we estimate that over 2.1 million unique patients were served across all countries receiving Americares donations in the U.S., Latin America, Sub-Saharan Africa, South Asia/East Asia/Pacific, Europe & Central Asia, and Middle East & North Africa. Based on the known number of course treatments donated to each region and extrapolating from our sample-level estimates of facility levels receiving donated medications, we estimated that 76% of the unique individuals served likely attended an outpatient healthcare facility and 24% likely attended an inpatient facility. This translated to a projected 5.1 million beneficiaries of the program, including an estimated 9% of beneficiaries with chronic conditions (N = 484,188) and 91% beneficiaries with acute conditions (N = 4,654,619). When all parameters in the model were simultaneously varied over a range of +/- 20% and run 10,000 times through the probabilistic sensitivity analysis, 95% credible intervals were simulated. Intervals for chronic medications tended have less uncertainty than acute medications, which tended to have wider variation.

## Discussion

Through analysis of Americares monitoring and evaluation data, we estimated the total number of individuals served by Americares medical donations during 2015 using an algorithm developed to translate course treatments to individuals. Table 1 indicates that the average number of drugs prescribed per patient visit ranges from 2.18 to 3.61, which is similar to estimates from other peer-reviewed articles. For example, the WHO reported an average number of drugs prescribed per patient encounter ranging from 1.3 to 4.4 between 1988 to 1992.[10,15] More recent articles from Nigeria, India, China, Sudan, Iran and other countries suggest a narrower average ranging from 2 to 3.68, which is very close to our estimated range.[16–25] Reports in the literature also indicate the average number of medicines dispensed in Ghana (4.3), Tanzania (2.2), El Salvador (2.2) and India (3.4) were comparable to our results of 3.12 (95% CI: 2.96–3.28), 2.49 (95% CI: 2.21–2.77), 2.18 (95% CI: 2.09–2.27) and 3.61 (95% CI: 3.49–3.73), respectively.[20,26] Our study further estimated that the average number of course treatments a patient takes home at each visit ranged from 1.25 to 3.62, depending on country and region.

Notably, we identified differences in the number of course treatments prescribed between our study countries, which may be due to differences in local physician prescribing patterns. For example, during our medical record review, we found that physicians in the Philippines were inclined to prescribe half of a total course treatments (e.g., 3 days of antibiotics instead of 7–14 days) and request that patients return for a follow-up visit to receive remaining doses; in contrast, physicians in the U.S. generally prescribed the full course treatments to patients during a single visit. The differences across countries in the average number of medications prescribed and the average number of course treatments per patient visit was largely influenced by the number of patients presenting with chronic vs. acute diseases, thereby affecting the revisit rate, clinical protocols for prescribing patterns, and quantities of medicines required for a full course of treatment. Identifying key parameters that likely differ across facility types and countries can help medication donation programs to better align donation strategies with local needs and improve their ability to accurately assess program impact.

Among the 10 facilities that participated in the Americares evaluation, we estimate that nearly 73,000 unique patients were provided with medications donated by Americares, yielding over 105,000 beneficiaries from the program. Scaled up to the country level, we estimate that over 700,000 unique patients were served, translating to 1.9 million total beneficiaries. A second extrapolation to the region level suggests that the 16.72 million total course treatments donated in 2015 served over 2.1 million unique patients, yielding 5.1 million beneficiaries. The number of chronic medications beneficiaries served compared to course treatments donated was proportionally lower than acute. This may be due to the fact that chronic diseases, such as diabetes or hypertension, are often are non-symptomatic and frequently remain undiagnosed. Acute conditions, on the other hand, may be apparent to patients resulting in greater frequency of doctors’ visits and subsequent dispensation of course treatments.

We should note that the facilities included in our sample store donated and self-procured medicines in same location; therefore, we were unable to ascertain whether the medicines prescribed at the time of our evaluation were donated or self-procured. However, we assume that both types of medicines were used indiscriminately and the source of medicines would not influence outcomes in terms of prescribing patterns, destruction rates, revisit rates or any other variables that feed into our algorithm. Therefore, we expect that the average number of course treatments per patient per encounter are not limited to assessments of donated medicines and can be applied more broadly to general prescribing patterns in each facility, country, and region.

Additionally, we chose to use the prescribed number of doses instead of a defined daily dose (DDD) to estimate course treatments in our algorithm. DDD is described by the WHO as the “assumed average maintenance dose per day for a drug used for its main indication in adults” and is often used to assess drug utilization.[27] While the DDD provides a standardized method for estimating trends in medication use across populations, it can vary greatly from the actual therapeutic dose prescribed and the number of different medications each individual patient obtains during a visit. In practice, prescribed doses vary based on a patient’s individual situation, potential for drug interactions, and other clinical indicators. For example, DDD for clindamycin (ATC code J01FF01) is 1.2 grams by oral administration. It does not represent the actual number of doses of clindamycin prescribed to an individual patient for their infection. Thus, using the number of prescribed medications in our calculation of course treatments supports a more accurate assessment donated medication use, strengthening the second stage of analysis for converting number of medicines to number of patients and beneficiaries.

Our study also identified important learnings and data quality issues regarding medication destruction rates that merit further attention if we hope to optimize the impact of medication donation programs. In our study, the main reasons for medication destruction cited by participants included expired drugs, mismatched supplies, damaged packages, and other supply chain issues. Additionally, most of the sample facilities do not have electronic or paper form inventory systems to track the inbound and outbound medicines and supplies. Therefore, the destruction rates used here were based on staff’s experiences and facility reports and may be an over- or under-representation of actual wastage. Quality data is essential to accurately monitor the outcomes of donated medicines and other medical supplies at the end user level. As such, we recommend that international donation organizations work together with facilities in recipient countries to strengthen inventory management systems to maximize the number of patients receiving potentially life-saving medicines and reduce wastage.

## Limitations

There are certain limitations to this study. First, medical records-based data collection in low-income countries pose data quality challenges. Most of the facilities did not have electronic medical record systems, and paper copies of health facility medical records were sometimes poorly organized, which could have implications for data quality. Additionally, inventory management systems at the facilities were sometimes limited. This led to difficulties in assessing medication destruction rates at the sample facilities, posing subsequent challenges for extrapolation to the country and region levels. Also, we had limited information on the proportion of donated medications that were destroyed, which constrained our ability to precisely estimate actual medication utilization. In lieu of destruction data from inventory systems, we relied on facility’s self-report, which may be biased. Future research should investigate methods for more precisely estimating destruction rates and the reasons that medications are destroyed. Additionally, improved inventory systems would support better monitoring of medication utilization, improve demand forecasting, and potentially reduce wastage. Data on facility and patient demographics, storage capacity, utilization, and specific medications prescribed were also not consistently available, but could improve efforts to ensure donations are highly relevant to recipient countries and optimize potential benefits of donation programs.

It was challenging to recruit facilities that maintained a complete medical record with prescription data to participate the study. We ultimately selected 3–8 local healthcare facilities in each country for participation, and this limited number of sample facilities could affect our extrapolation to the country and region levels. As such, we made assumptions and extrapolated beyond the sample data to estimate the program impact at the country and region levels. Utilization and prescription patterns at the facilities included in our sample may or may not be representative of facilities across a particular country or region. Prescription patterns can be influenced by regional epidemics and disease prevalence, in addition to medical education and patient preferences. Additionally, country and region-level estimates are projections and should be interpreted with consideration of the data limitations. To more accurately assess country- and region-level outcomes, a more rigorous, large- scale study including a broader selection of facilities and countries is needed to further examine our algorithm and determine the generalizability of its application to other settings and patient populations. However, our study collected primary data from individual facilities receiving donations from the program and reflected prescribing practices on-the-ground. While we extrapolated beyond the sample to project the full impact of the program, we conducted a robust uncertainty analysis to estimate the reliability and range of our projections.

Finally, while a medical donation program’s success in reaching target populations should be a key indicator of performance, other outcome measures have also been proposed[28]. Examples of outcome measures that were beyond the scope of this evaluation include reductions in morbidity, disability-adjusted life-years, and mortality. While well intentioned, the goals of international donor programs do not always align with the needs, storage capacity, or supply chain resources required to manage a large influx of medications. This can create additional challenges for already overburdened health systems. In addition to the number of patients receiving treatment, future impact evaluations should monitor the potential waste created, human capital lost, and any health systems bottlenecks created through medical donations.

## Study implications

This study provides a methodology for other medical donation programs seeking to estimate their impact and global reach. The Partnership for Quality Medical Donations (PQMD), the main alliance of companies, NGOs, and non-profits facilitating medical donations, has called for better monitoring and evaluation of program impact and outcomes.[29] To our knowledge, this is the first study to provide both a strategy for estimating impact and algorithm for translating medications donated into patients served and total beneficiaries.[20,29] In doing so, we provide our peers in the medical donation space with a blueprint for calculating the number of patients served by their own programs. Understanding this core program outcome may support better allocation of donated resources and improve coordination efforts between donors and recipients.

We believe that collecting and analyzing data to assess the medicine donation process from donor inventory to patient benefit is a critical first step to better evaluating the value that medical donation programs provide. However, data tracking between the donor and recipients need to be improved to be able to assess impact with more rigor. First, improving inventory management systems for the end users in recipient countries is key to monitoring the use of and need for medicines and supplies. Health facilities, patients, and the international medical donation community would all benefit from improved data quality that would come from better inventory management systems. Second, at the country and region level, national pharmaceutical surveillance systems could provide a structure in which medication and utilization data is better monitored. Finally, the algorithm used in this study should be validated through a larger sample of facilities with a more rigorous sampling process.

This novel approach of monitoring international medical donation provides an improved approach for humanitarian medical professionals and nonprofit organizations to determine the reach of international medical donation activities.

## Supporting information

S1 Appendix (DOCX)

S2 Appendix (DOCX)

S1 Dataset (XLSM)
