# Supplementary material for: Translating medicines to patients: A novel methodology for quantifying the global medical supplies and donations program
Source: PLoS One. 2018 Nov 2;13(11):e0206790. doi: 10.1371/journal.pone.0206790 (PMC6214557; doi:10.1371/journal.pone.0206790)
Supplement: S1 Appendix — (DOCX) [file pone.0206790.s001.docx]

**Appendix 1. Americares Standard Operating Procedures for Calculating Course Treatments**

**Calculating Product Donation Course Treatments**

**INTRODUCTION:** AmeriCares employs a detailed methodology to qualify and quantify a diverse array of donated products that are delivered to our global partners, healthcare institutions and individual beneficiaries. The steps involved in determining course treatments are part of the evaluation process for accepting and allocating various pharmaceuticals, nutritional preparations, medical supplies and medical consumables. Identifying the number of course treatments allows AmeriCares to maximize product utilization, and is both relevant and important in measuring the actual impact of individual donations.

Course treatment calculations provide estimates of the potential numbers of patients that will benefit from a specific donation within a specified time frame. The calculations are based on each product’s therapeutic characteristics such as primary indication, average daily dose, duration of treatment and the age of the recipient patient population. The *precise* number of patients treated, or the number of facilities supported can differ slightly from the projected numbers. However, this tool is intended to aid in the product acceptance process, and guide the allocation of products to the most appropriate healthcare facilities.

Both the packaging and the product’s primary indication are the basis for nearly all course treatment calculations. Listed below are those steps used to determine the number of course treatments of pharmaceuticals and nutritional preparations. *

1. The product’s therapeutic uses and primary indications are specified
2. The duration of therapy for the primary indication is distinguished between acute, long-term acute and chronic therapy:
3. **Acute therapy** occurs when the recommended therapeutic regimen is completed in thirty days or less.
4. **Long-term acute** **therapy** occurs when the recommended acute course treatment is more than 30 days (one month). These are calculated using the chronic course treatment calculation method, and expressed as the number monthly course treatments, although a course of therapy may be longer than one month.
5. **Chronic therapy** occurs when the recommended therapeutic regimen last longer than 30 days. The calculation defines the monthly duration of treatment as 30 days.
6. The average daily dose for the primary indication is then multiplied by the number of days of therapy providing the amount of product needed for each course treatment.
7. The amount of packaged product is then divided by a number that represents the total amount of product required for each course treatment. The resulting value is the number of course treatments available per package.
8. In certain cases the manufactures’ packaging will determine the course treatment numbers. Some products are manufactured for individual patient use; one package is equivalent to one treatment course. Other products are package to be dispensed to several recipients.

**Examples of Calculation Methods for Course Treatments:**

1. **Calculation Method for Acute Course Treatments:** When therapeutic indications entail treatment duration less than or equal to 30 days the total number of doses is based on the primary indication and the average dosing regimen for specified indication. For example the number of doses per day is multiplied by the total number of treatment days. Normally these products are used to treat acute diseases/disorders/symptoms and prescribed for a specified time.
   1. **Clarithromycin, 500 mg tablet, 60’s:**
      1. Therapeutic indication for lower respiratory tract infections,
      2. Average adult daily regimen is 500 mg (one tablet) twice daily
      3. Average duration of treatment 10 days
      4. One tablet twice daily X 10 days = 20 tablets/course treatment
      5. Course treatment per bottle is: 60 ÷ 20 = 3 course treatments/bottle
   2. **100 ml bottle of Amoxicillin 400 mg/5 ml + Potassium clavulanate, 57 mg/5 ml, Powder for oral suspension**
      1. Therapeutic indication is respiratory tract infections
      2. Average daily pediatric dose: one teaspoonful (5 ml) twice daily
      3. Average duration of treatment 10 days
      4. Total amount of drug/course treatment: 5 ml X 2 X 10 days = 100 ml.
      5. Course treatment per bottle is: 100 ml ÷ 100 ml = 1 course treatment.
2. **Long-term acute:**  When the recommended duration of treatment for the acute primary indication is more than 30 days, the chronic course treatment calculation method is used and the result is expressed as the number of monthly course treatments. The total amount of product required for of the complete course of treatment is used for the calculation of course treatment number.
   1. **Lansoprazole, 30 mg capsule, bottle of 60’s**
      1. Primary indication is erosive gastroesophageal reflux disease (GERD)
      2. Average adult daily regimen is one capsule once daily
      3. *Average* duration of treatment 8 weeks or two months: (*The duration is > 30 days therefore use chronic course treatment methodology*)
      4. Total amount of drug/30 days = 1 cap X 30 = 30 caps
      5. Monthly Course treatments per bottle are 60 ÷ 30 = 2 monthly course treatments per bottle. [Note: The bottle actually contains one *average* course treatment that may last approximately full 2 months.]
3. **Calculation Method for Chronic Course Treatments:** When therapeutic indications entail a treatment duration greater than 30 days we express the number of treatments as “months of treatment” or “monthly course treatments.” By establishing this constant unit of measure, “one month of treatment,” AmeriCares is factoring in practical considerations for how prescriptions are actually dispensed - typically in one-month increments. This approach also allows for a calculation that is not distorted by different expiration dates and treatment durations (e.g. 6 months vs. lifetime). Two examples follow:
   1. **Paroxetine, 20 mg, bottle of 100’s**
      1. Primary indication is chronic management of symptoms of depression
      2. Average daily dose regimen is 20 mg once daily.
      3. Duration of treatment 30 days
      4. One tablet once daily X 30 days = 30 tablets/course treatment
      5. Monthly course treatment per bottle = 100 ÷ 30 = 3.3 monthly course treatments per bottle.
   2. **Budesonide, 160 mcg /1 actuation + Formoterol fumarate 4.5 mcg/1 actuation, Pressurized inhalation, powder (120 actuations per container)**
      1. Primary indication is chronic management of asthma
      2. Average adult daily regimen two actuations taken twice daily
      3. Duration of treatment 30 days
      4. Two actuations twice daily X 30 days = 120 actuations/course treatment
      5. Monthly course treatment per container is 120 ÷ 120 = 1 monthly course treatment
4. **Single-Use Containers:** These products are packaged specifically for use as one course treatment even though these items may be used more than one time or a single day. Only one patient will receive one package for the entire course treatment. Examples:
   1. **One tube of 1% Silver Sulfadiazine (50 gm) is considered one course treatment**
   2. **10 tubes of 1% Silver Sulfadiazine (50 gm) are calculated as 10 course treatments.**
   3. **6 bottles of Gentamicin Ophthalmic Solution (5 ml) are calculated as 6 course treatments.**
5. **Multi-Use Containers:**  These products contain several acute or chronic course treatments in each package. The course treatment numbers are determined by product and on a case-by-case basis. They depend upon the specific context in which the item will be used. The package may be used for the treatment of one patient or more. Examples listed below:
   1. **One bottle - 90 tablets of Lisinopril, 20 mg**
      1. Primary indication is the chronic management of hypertension, a chronic disease process often treated over many years.
      2. Average daily adult dose regimen will be one tablet daily for a patient receiving this dose of lisinopril.
      3. Duration of treatment for chronic regimens is calculated in 30-day courses.
      4. Course treatment calculation is daily dose for primary indication multiplied by duration of treatment (chronic disease, use 30 days). 1 tab X 30 days = 30 tabs/monthly course treatment).
      5. Bottle of 90 tabs will provide one patient with 3 monthly courses of antihypertensive therapy or 3 patients with one monthly course treatment of therapy. (*90 ÷ 30 = 3 monthly course treatments/bottle*)
   2. **One package - 18 tablets of Azithromycin, 250 mg:**
      1. Primary indication is Rx of certain susceptible bacterial infections.
      2. Typical anti-infective dosing of azithromycin, 250 mg, is two tablets on the first day, then one tablet daily for the next four days, which means 6 tablets are needed per course treatment.
      3. The amount in each package (18 tabs) divided by the total number of tablets per treatment course (6 tabs). 18 ÷ 6 = 3 course treatments per package.
      4. This medicine is used acutely, often for respiratory diseases, and the potential number of patients that could be treated would be 3, or 3 course treatment.

**AmeriCares Methodology for Calculating Courses of Treatment**

Original version: January 29, 2008 Revised: February 1, 2011

*Course treatment calculations are based upon the most practical, predictable drug usage for packaging and doses supplied, in addition to a primary indication and the specified population (adult vs pediatric) for drug use. For example, the WHO defined daily dosage (DDD) for an anti-hypertensive ACE inhibitor might be 10 mg per day for drug A. This drug may be supplied by the manufacturer or donor in tablets of 5, 10, 20 and 40 mg. If we are calculating the number of monthly course treatments for a bottle of thirty 40 mg tablets of drug A it does *not* represent 4 monthly course treatments. It represents *a single monthly course treatment for a patient who is receiving the higher. 40 mg dose, of drug A as part of their anti-hypertensive regimen.* This, in fact, is how it will be used. It will not be split into four pieces. For products such as topical antibiotics and corticosteroids for dermatological use, each tube will be used by one patient for a single course treatment as described above.
