# Supplementary material for: Translating medicines to patients: A novel methodology for quantifying the global medical supplies and donations program
Source: PLoS One. 2018 Nov 2;13(11):e0206790. doi: 10.1371/journal.pone.0206790 (PMC6214557; doi:10.1371/journal.pone.0206790)
Supplement: S2 Appendix — (DOCX) [file pone.0206790.s002.docx]

**Appendix 2. Key Formulas in Americares “Course Treatment to Beneficiaries” Algorithm**
